# The Prevention of Abuse at the Hospitals

**Published:** 1907-07-20

**Authors:** 


### THE PREVENTION OF ABUSE AT THE HOSPITALS.

### I.—WHAT THE ROYAL FREE HOSPITAL IS DOING.

The prevention of hospital abuse is a problem which institutional authorities have had before them, it may be said, almost from the very beginning of the establishment of these charities. In the early years of the last century, when the number of out-patients attending the London hospitals was small compared with the number at present on the books, the practitioner not attached to the hospital complained that "such institutions stole away, by gratuitously and unnecessarily treating patients well enough able to afford the charges of a private doctor, most of the medical man's custom." Since then the evil, and the problem of dealing with it, have become most serious and formidable. Some of the smaller institutions have attempted a method of weeding out, a kind of selection of out-patients, with, in certain cases, conspicuous success; and as these endeavours to reform an evil which is one of the worst of our present hospital system are not only interesting but instructive, as samples of what might be done were the matter seriously and systematically attended to, we purpose giving a brief description of the various methods adopted for checking hospital abuse.

The First Hospital Almoners.

To the Royal Free Hospital, Gray's Inn Road, belongs the credit of being the first institution which seriously set itself to consider a means by which the patient able to afford private treatment could be eliminated from the mass of hospital out-patients, just as to Marsden, its founder, belongs the credit of having been one of the first to preach the doctrine of "discriminating charity." In 1894 the authorities of this hospital instituted a system of inquiry into the "means" of certain of the out-patients This, the beginning of the present system of almoners, was necessarily incomplete and fragmentary. Comparatively few cases were dealt with, but enough was done to bring into prominence the fact that there were abuses, and that something was necessary to check these. For the first two years, the work of inquiry was carried on amidst great difficulties—not only departmental, but arising from objections raised by the medical and surgical staff, who complained that "such a system tended to limit the number of out-patients and consequently to militate against the excellence of the teaching material." Still, through the system of almoners which was instituted by this hospital and has since been adopted, we are glad to say, by other institutions, the beginning of a greater and more systematic arrangement was made.

The Duties of the Almoners.

There are now two "Lady Almoners," one in charge of the out-patient department and the other solely concerned with in-patient work. Attached to these are a few volunteer helpers. In many respects the duties of the almoners are similar. They have to inquire into the merits of each case, to find out whether the patient is in a position to contribute towards the expense of treatment, to help out of a fund specially provided for such purposes those who are absolutely destitute, and to report to the staff instances in which, in their opinion, patients are abusing the benefits of the charity. In the out-patient department all patients are, as far as practicable, seen by the almoner with a view to the elimination of unsuitable applicants, as well as to render adequate the treatment given. Nor does the almoner's work stop here, for it is her duty to interest herself in individual cases and to endeavour to find permanent work, underfavourable conditions, for convalescent patients out of employment. She has to try to persuade patients who are able to do so to join a good Friendly Society or Provident Dispensary, and in this manner she inculcates thrift. The work that is entailed by all this is by no means light; it puts a strain on the almoner and on her individual assistants which is bound to increase as the hospital broadens its scope of usefulness and the attendance of out-patients becomes greater.

The Out-patient Almoner.

The out-patients attend in the morning at the out-patient entrance in Gray's Inn Road. There they are seen by the senior resident medical officer and the senior house-surgeon. These officers treat the minor out-patients—that is to say, cases which, in their opinion, need not go before any of the visiting staff. To each minor out-patient a pink slip is given of which the following is a copy :—

ROYAL FREE HOSPITAL.
Gray's Inn Road, W.C.

*Enquiry Officer* .................................... *No.* ...........

Minor Out-patients.
For further treatment the patient must attend in the Out-patient Department at 1 p.m.
on any day except Monday.
(Admittance by Out-patient Entrance only.)

*Name* Edward Jones..........................................................
*Age* 16. *Disease* Abscess................................................
*Date* 16/9/06 *Med. Officer* O. O. S.................................

*Notes*—
Treatment prescribed.

Almoner's report on case.

With this slip the patient attends the dispensary and receives the medicine prescribed. He is then sent to the almoner's office, where he is personally seen and interrogated by Miss Brimmel, out-patient lady almoner, or her assistant. This interrogation is searching and definite. He is asked, if an adult, whether he is married, whether employed, and if so, what his earnings are, what his average expenditure amounts to, and, finally, whether he is able to pay anything at all towards the expenses of treatment. Generally, it is found that patients are in a position to contribute towards payment for any surgical appliances they may need. In that case, they sign a paper agreeing to pay so much a month towards the cost of splints, leg-irons, etc., provided by the hospital. This agreement serves its purpose well. Its object is to make patients sensible of the fact that gratuitous help can only be given, in justice, to those who are absolutely unable to help themselves. These latter are treated with the greatest consideration. Out of the Dresden Fund assistance is given them in money; and their convalescence is helped by sending them to a home, the expenses of such a holiday being borne by the fund. This applies particularly to serious cases, which may be grouped under the heading of in-patients, and with these we shall deal presently. When the almoner finds that the minor out-patient is in a position to afford private treatment, or that from other causes his attendance is undesirable, in so far as it constitutes an abuse of the charity, she withdraws his slip of pink paper. He is allowed to get his medicine once—as a matter of fact, he has already received it before he enters the almoner's office—but he is not permitted to attend again, nor is he allowed to continue to receive treatment or medicine. He is summarily weeded out—a loss to the hospital of which no one need complain. Probably he goes to another hospital, where no such system is in force, and there receives medical help and medicine at the expense of more deserving and less impudent patients.

In the case of major out-patients, who are seen by a member of the visiting staff, the almoner's privilege is limited to an investigation into the circumstances of the cases, the results of which she reports to the visiting staff. If she finds that the applicant is undeserving of charitable help, or is only using the hospital as a refuge, she notes the fact for the physician or surgeon in charge of the case, and he then exercises his discretion in regard to it. Generally, it is satisfactory to note, the visiting staff are ready to support the almoner by acting on her suggestions, and, so far, the system has worked admirably.

The In-patient Almoner's Methods.

The in-patient almoner, Miss Lucy, visits and reports on all cases admitted to the wards. "I make it a rule," she said, "to assume that each patient is able to pay something towards the cost of any apparatus provided for him." Only when a patient alleges that he is unable to contribute does the in-patient almoner investigate to the full his claims upon the hospital. The valuable help given by the Dresden Fund is of incalculable assistance to the in-patient almoner in assisting really destitute and deserving patients. In each case the assistance given by the fund is limited to £10—a limit which, under no circumstances, may be overstepped. Last year no fewer than 362 patients were so assisted by the fund, every case having been systematically investigated and found thoroughly eligible.

It is easy to see that such a system of almoners as this of the Royal Free Hospital must be of great and much-needed assistance to any charitable institution which desires to dispense its bounty to the truly deserving and distressed applicant, and to draw a sharp line of demarcation between such patients and those who abuse the charity by begging assistance while they are in a position to call in the help of a private doctor.

Details of In- and Out-Patient System of Inquiry.

The almoner exercises her discretion in dealing with individual cases. In one case a man may be earning 23s. to 25s. a week, and that fact may seem sufficient to warrant the assumption that he will be able to pay for his child's leg-iron. On investigation, however, the almoner finds that he has only been earning that sum for a few weeks, having been out of employment for a considerable length of time, and that during his out-of-work time he has been very unfortunate. All these circumstances will have to be, and are being, taken into consideration. That great crux of hospital relief questions—maternity cases— does not concern the almoner directly. It is only recently that the Royal Free has commenced extern maternity work, and destitute cases in the district are reported to the relieving officer. "We discourage giving such cases relief," say the almoners, "for we do not think it is right to add an inducement to the husbands not to provide for them. As a matter of fact, however, we get few of these cases; most of them go to neighbouring charities—King's and University, for instance, who are encouraging such cases for teaching purposes." One naturally wishes to know how the system works so far as the patients themselves are concerned. "Excellently," says the lady almoner. "Patients do not resent our interest in their affairs; they are only too eager to tell us their circumstances. Nor do they in general resent being asked to contribute. When the position is clearly put before them, and when they are made to see that we are not really unfriendly, they are quite content to pay their small shares. Now and then we get an exception—the man who declares that he 'is going to write to the paper' about our methods—but these are rare." Begging relief is discouraged as much as possible; for that reason the almoners look askance at the Surgical Aid Society. But other bodies are actively used. Thus during last year the following charities were helpful in assisting the almoners: Home for the Dying (advanced cancer cases), James Keats' Home (a so-called "Rest Home"), House of Charity, Truss Society, N.S.P.C.C. ("We dislike reporting to this Society," says Miss Brimmel, "but in a few cases, unfortunately, where the children suffer through the wilful neglect of the parents, we have been obliged to do so"), Metropolitan Association for Befriending Young Servants, Special Hospitals, Mildmay Mission, St. Albans' Mission, Salvation Army, Metropolitan Nursing Association, Great Northern Hospital. At the same time patients are earnestly asked to consider the advisability of joining a good provident society, and the almoners make a point of distributing the cards of some of these societies, which set forth in detail the advantages to be gained by becoming a member.

[The Editor will be glad to hear from the secretaries of any institutions where any system of inquiry is in force, with reference to details of the methods adopted.]

